# The use of longitudinal CT-based radiomics and clinicopathological features predicts the pathological complete response of metastasized axillary lymph nodes in breast cancer

**DOI:** 10.1186/s12885-024-12257-y

**Published:** 2024-05-01

**Authors:** Jia Wang, Cong Tian, Bing-Jie Zheng, Jiao Zhang, De-Chuang Jiao, Jin-Rong Qu, Zhen-Zhen Liu

**Affiliations:** 1 Department of Breast Disease, Henan Breast Cancer Center, The Affiliated Cancer Hospital of Zhengzhou University & Henan Cancer Hospital, 127 Dongming Road, Zhengzhou, Henan 450008 China; 2 Department of Radiology, The Affiliated Cancer Hospital of Zhengzhou University & Henan Cancer Hospital, 127 Dongming Road, Zhengzhou, Henan 450008 China

**Keywords:** Breast cancer, Axillary lymph node, Radiomics, Computed tomography, Neoadjuvant chemotherapy, Pathological complete response

## Abstract

**Background:**

Accurate assessment of axillary status after neoadjuvant therapy for breast cancer patients with axillary lymph node metastasis is important for the selection of appropriate subsequent axillary treatment decisions. Our objectives were to accurately predict whether the breast cancer patients with axillary lymph node metastases could achieve axillary pathological complete response (pCR).

**Methods:**

We collected imaging data to extract longitudinal CT image features before and after neoadjuvant chemotherapy (NAC), analyzed the correlation between radiomics and clinicopathological features, and developed models to predict whether patients with axillary lymph node metastasis can achieve axillary pCR after NAC. The clinical utility of the models was determined via decision curve analysis (DCA). Subgroup analyses were also performed. Then, a nomogram was developed based on the model with the best predictive efficiency and clinical utility and was validated using the calibration plots.

**Results:**

A total of 549 breast cancer patients with metastasized axillary lymph nodes were enrolled in this study. 42 independent radiomics features were selected from LASSO regression to construct a logistic regression model with clinicopathological features (LR radiomics-clinical combined model). The AUC of the LR radiomics-clinical combined model prediction performance was 0.861 in the training set and 0.891 in the testing set. For the HR + /HER2 − , HER2 + , and Triple negative subtype, the LR radiomics-clinical combined model yields the best prediction AUCs of 0.756, 0.812, and 0.928 in training sets, and AUCs of 0.757, 0.777 and 0.838 in testing sets, respectively.

**Conclusions:**

The combination of radiomics features and clinicopathological characteristics can effectively predict axillary pCR status in NAC breast cancer patients.

## Introduction

Breast cancer patients with axillary lymph node metastasis often need neoadjuvant chemotherapy (NAC). For NAC patients, pCR means no residual invasive carcinoma in either the primary breast tumor lesion or the positive axillary lymph node. Previous studies showed that approximately 20–40% of NAC patients with axillary lymph node metastasis could achieve axillary pCR [1], with higher proportions observed in triple-negative and HER2-positive patients [2]. Achieving axillary pCR holds greater significance compared to the primary lesion for evaluating the patient’s prognosis [3]. However, NAC patients with axillary lymph node metastasis generally require direct axillary lymph node dissection (ALND) after completing NAC [4, 5]. This procedure can lead to complications such as impaired upper limb function, numbness, pain, and even lymphedema [6–8]. Therefore, for patients who might achieve axillary pCR through NAC, the potential benefits of ALND may be outweighed by the risk of surgical complications. These patients should be considered for surgery de-escalation to avoid these complications [9]. The key issue lies in how to determine axillary pCR before surgery. If a noninvasive approach can accurately assess the axillary lymph node status before surgery, axillary pCR patients could even be exempted from axillary surgery. It may hold significant decision-making implications for surgeons.

Traditional prediction models for axillary pCR often rely solely on clinical and pathological features and their performances are often unsatisfactory [10, 11]. One possible reason is that these models lacked longitudinal information on changes in axillary lymph node status after NAC. Additionally, even if post-NAC information was included, there may still be a disparity between the predicted model and clinical application if the model does not encompass the entire axillary situation [12, 13]. These studies only included cN1 to cN2 stage patients, without considering information about infraclavicular lymph node metastasis. They were limited to providing a more accurate and comprehensive description of the axilla. If data encompassing the entire axillary region can be obtained, the prediction model performance could be significantly improved.

As an interdisciplinary field combining medical imaging and computer vision, radiomics is gradually playing a significant role in assisting clinical diagnosis and treatment strategies. It also has been used to assess axillary lymph node status in breast cancer patients [14–16]. However, these studies still only focused on one positive lymph node as a target rather than the whole axilla.

In this retrospective study, we considered the entire axilla as the region of interest (ROI) for radiomics analysis and incorporated both pre-NAC and post-NAC computed tomography (CT) images to add longitudinal information. We aimed to assess the feasibility of predicting axillary pCR after NAC using radiomics with clinical and pathological information.

## Materials and methods

### Patient enrollment

**Fig. 1:**
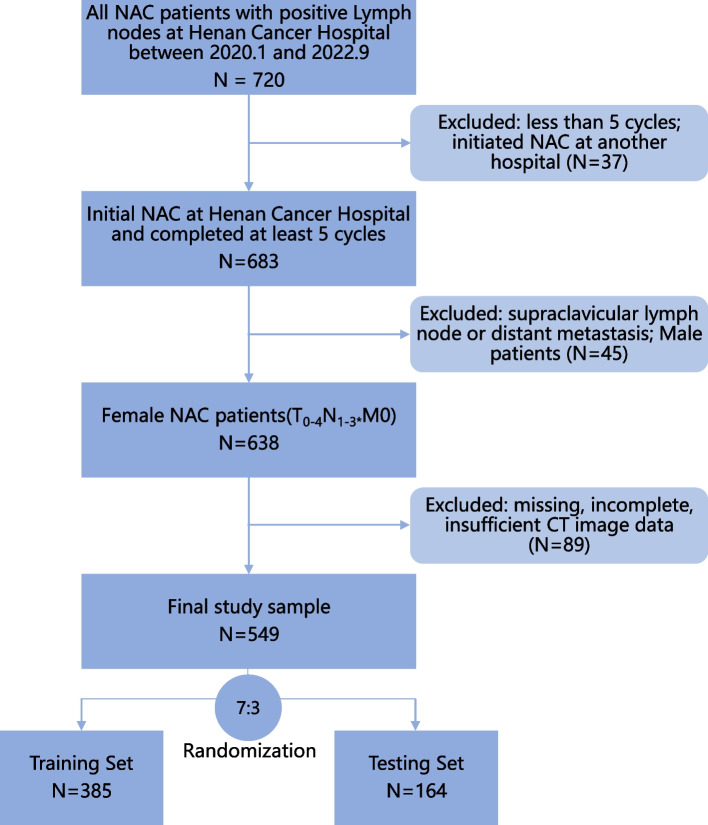
Patient recruitment process and study workflow. *:N3 only includes infraclavicular lymph node metastasis

This study initially included 720 breast cancer patients who underwent NAC followed by surgery at Henan Cancer Hospital between January 2020 and September 2022. All patients had confirmed axillary lymph node (including infraclavicular lymph nodes) metastasis through either core needle biopsy or pathological consultation before NAC. Enrollment and exclusion criteria for analysis is shown in Fig. 1. The required imaging data and clinical pathological information were obtained through the Picture Archiving and Communication System (PACS) and Electronic Medical Records system. This retrospective study obtained approval from the institutional ethics review board (No. 2017407). Informed consent had been obtained from each patient at the time of the examination for imaging and clinical data.

All included patients underwent pre-NAC clinical staging according to the eighth edition American Joint Committee on Cancer (AJCC) breast cancer staging system. Expression of ER, PR, HER2, and KI67 index were detected by immunohistochemistry. When HER2 expression was 2 + , HER2 gene amplification was determined by fluorescence in situ hybridization (FISH). Then patients were classified into Luminal A/ B (HER2 -), Luminal B (HER2 +), HER2 enriched, and Triple Negative subtypes. All patients underwent levels I-II ALND, and N3 (infraclavicular lymph node metastasis) patients also underwent additional level III dissection. The dissected axillary lymph nodes were analyzed by pathologists, and the absence of invasive cancer residue in all lymph nodes was considered axillary pCR; otherwise, it was considered non-pCR.

### Treatments

Patients with different molecular subtypes received corresponding NAC regimens. For HER2 positive patients, a dual-targeted chemotherapy regimen containing taxanes, trastuzumab, and pertuzumab was administered. For luminal subtype (HER2 negative) and triple negative patients, chemotherapy regimens combining taxane with anthracycline or with platinum drugs were administered. Treatment changes due to progressive disease (PD) during NAC were considered *regimen change*, while simple deletion of chemotherapy drugs or cycles were still considered the original regimens.

### CT scan and image processing

Patients should undergo routine chest CT scans before and after NAC. All CT scans were performed following a standardized protocol on one of the three CT systems: Brilliance iCT scanner (Philips Healthcare), uCT 760 (United Imaging), and SOMATOM Perspective (Siemens Healthineers). The main scanning parameters were as follows: tube voltage = 120 kV, automatic tube current modulation (30–70 mAs), pitch = 1.0–1.5, matrix = 512 × 512, and field of view 350 mm × 350 mm. The slice thickness was from 0.625 mm to 1.25 mm. The acquired mediastinal window CT plain scan images were in DICOM format series images. The dicom2nifti library (python 3.7) was used to batch convert DICOM series images into *nii.gz* files with the image resampling unified to 1 mm, 1 mm, and 1.25 mm (thickness). Then, two senior radiologists used 3D slicer software (5.0.2) to draw the entire axilla of the affected side as ROI and saved it as a MASK file. Then, the PyRadiomics module was used to batch-extract radiomics features from nii.gz files and their matched MASK files [17]. To obtain more derived features, we also used wavelet, gradient, and Laplacian of Gaussian (LoG) filters. In particular, as an edge enhancement filter, the LoG filter can highlight different image textures by adjusting the parameter Sigma. The roughness of the image texture is inversely proportional to the value of Sigma. In this study, the parameter range of Sigma was set from 2 to 8 to obtain texture features with different levels of fineness (Sigma = [2,3,4,5,6,7,8]). 3190 radiomics features can be obtained from each patient undergoing two CT scans. Inter-observer and intra-observer consistency was performed by analyzing all radiomics features extracted based on intra and interclass correlation coefficients (ICCs). ICC > 0.8 suggested good agreement.

### Statistics

**Fig. 2:**
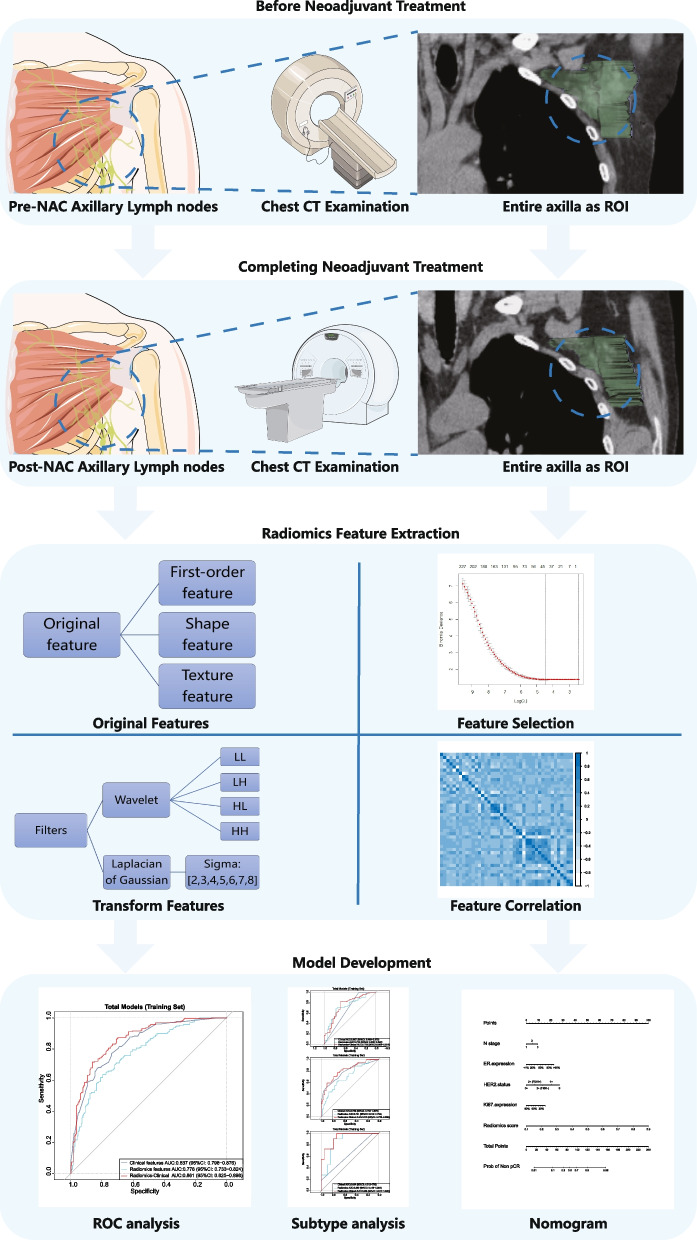
The study workflow of necessary steps in predicting axillary pCR after NAC. The entire axilla was drawed as ROI (blue dashed circle)

Patients were divided into a training set and a testing set at a ratio of 7:3. Then they were divided again into two groups according to whether they obtained axillary pCR. We employed R 4.2.1 and SPSS 26.0 (IBM, USA) for statistics. Continuous variables are described as the mean ± SD. The comparison between the two groups was performed using χ^2^ test for categorical variables and independent t-test or Mann–Whitney U test for continuous variables. The obtained radiomics features were screened for significantly different features through Student’s t-test and least absolute shrinkage and selection operator (LASSO) regression algorithm [18]. Feature importance was subsequently evaluated using the explain function (DALEX library, R software 4.2.1). The correlation between radiomics features and baseline clinical features was analyzed. Then, we built a pure radiomics features model and developed a possible prediction score (*Radiomics score*) using this algorithm. Radiomics score was combined with these clinical features to build an integrated clinical-radiomics model through logistic regression (LR radiomics-clinical combined model). Clinical features model was also constructed by logistic regression. Receiver operating characteristic (ROC) curves were drawn for each model. Bilateral *P* values < 0.05 were regarded as significant. Subtype analyses were the same as above. Finally, a nomogram was created using the rms library (R software). Calibration curve were also generated to examine the performance of the nomogram. The study workflow is detailed in Fig. 2.

## Results

### Baseline clinicopathological characteristics

A total of 549 cases were obtained after screening, including 290 axillary pCR patients and 259 non-pCR patients. Then these patients were randomly divided into training set and testing data in the ratio of 7:3 (see Fig. 1). Table 1 shows the clinicopathological characteristics of the patients in the training set (*n* = 385) and the testing set (*n* = 164). There were significant differences between the two groups, such as clinical N stage, molecular subtype, IHC markers, and treatment regimen, while there were no significant differences in age, height, weight, or primary tumor T stage.

**Table 1:** Baseline characteristics of the axillary pCR and non-pCR groups

Clinical features	Groups	Training Set		*P*	Testing Set		*P*
		**pCR (*N* = 195)**	**Non-pCR(*N* = 190)**		**pCR (*N* = 95)**	**Non-pCR(*N* = 69)**	
Age (years)^a^		49.7(± 9.2)	48.8(± 9.6)	0.350	50.1(± 9.4)	47.0(± 10.9)	0.055
Height (cm)^a^		159.7(± 12.0)	159.5(± 7.2)	0.822	159.6(± 5.1)	160.2(± 4.2)	0.396
Weight (kg)^a^		63.1(± 9.1)	64.3(± 11.4)	0.236	61.9(± 9.1)	62.2(± 9.5)	0.798
T stage^b^	0	1	1	0.818	0	0	0.770
	1	13	15		5	4	
	2	151	138		76	51	
	3	22	24		11	10	
	4	8	12		3	4	
N stage^b^	1	106	96	0.301	59	32	0.011
	2	38	31		21	12	
	3	51	63		15	25	
Subtype^b^	Luminal A/ B (HER2 -)	40	109	< 0.001	9	39	< 0.001
	Luminal B (HER2 +)	75	48		31	16	
	HER2 enriched	62	16		39	6	
	Triple Negative	18	17		16	8	
ER expression^c^		5 (0, 80)	90 (30, 95)	< 0.001	0 (0, 20)	90 (10, 95)	< 0.001
PR expression^c^		0 (0, 20)	35 (1, 80)	< 0.001	0 (0, 7.5)	40 (0, 85)	< 0.001
Ki67 index^c^		50 (40, 70)	40 (30, 60)	< 0.001	50 (40, 70)	40 (30, 70)	0.151
HER2 status^b^	0	11	20	< 0.001	6	10	< 0.001
	1 +	27	34		8	11	
	2 + (FISH-)	20	72		11	26	
	2 + (FISH +)	19	32		13	13	
	3 +	118	32		57	9	
Treatment^b^	anthracycline and taxane	48	120	< 0.001	18	43	< 0.001
	dual-targeted drug	137	62		70	21	
	taxane and platinum	10	4		6	3	
	regimen change	0	4		1	2	

^a^independent T test

^b^χ^2^ test

^c^Mann-Whitney U test

### Correlation analysis of radiomics and clinicopathological features

**Fig. 3:**
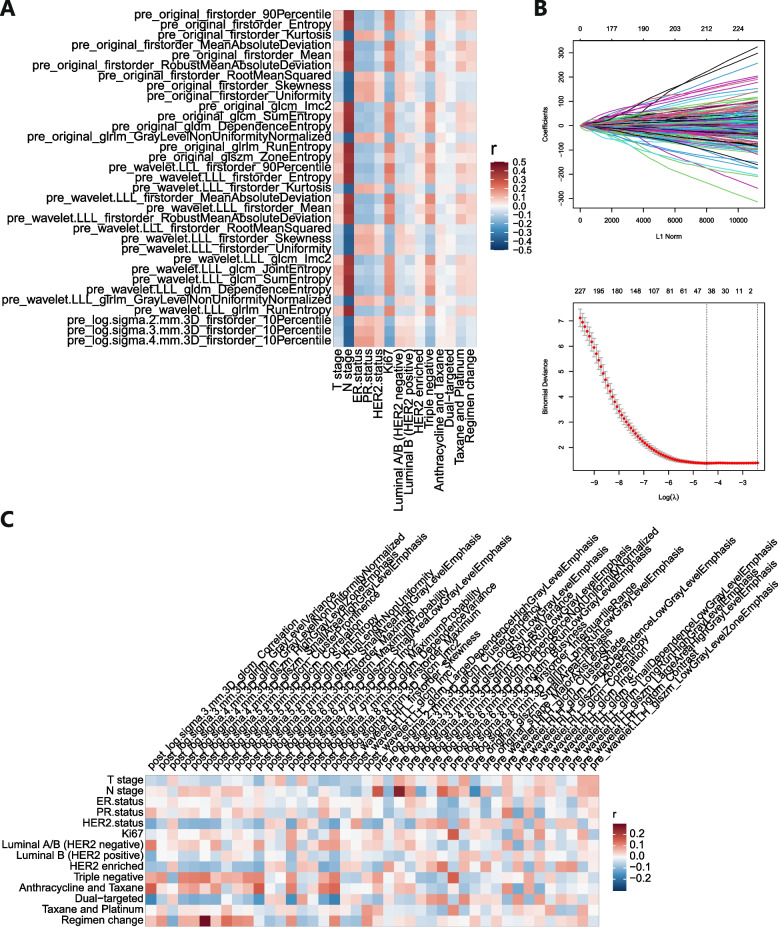
Correlation analysis before and after feature screening. **A** The relation between radiomics features and clinicopathological features before LASSO regression screening. **B** Feature coefficients corresponding to the value of parameter λ. The most valuable radiomics features were screened out by tuning λ using LASSO regression with 10-fold cross-validation. **C** The relation between screened radiomics features and clinicopathological features

Then, from the 3190 obtained features, we found that the correlation coefficients of 33 radiomics features with clinicopathological features were greater than absolute correlations of 0.35. These features had the strongest correlation with the clinical N stage and were all pre-NAC features (Fig. 3A). Then, these 3190 features were screened through t-test and LASSO regression. A total of 42 radiomics features were obtained and significantly related to axillary pCR (Fig. 3B), including 21 pre-NAC features and 21 post-NAC features. Pre-NAC features were mainly wavelet (*n* = 10) and LoG (*n* = 9) filter-derived features; post-NAC features were mainly LoG (*n* = 18) filter-derived features. These 42 features had no intersection with the previous 33 features that were significantly related to the clinical N stage. Their correlation with baseline clinical features was much lower (generally between -0.3 and 0.3, Fig. 3C).

### Performance of clinical and radiomics models

**Fig. 4:**
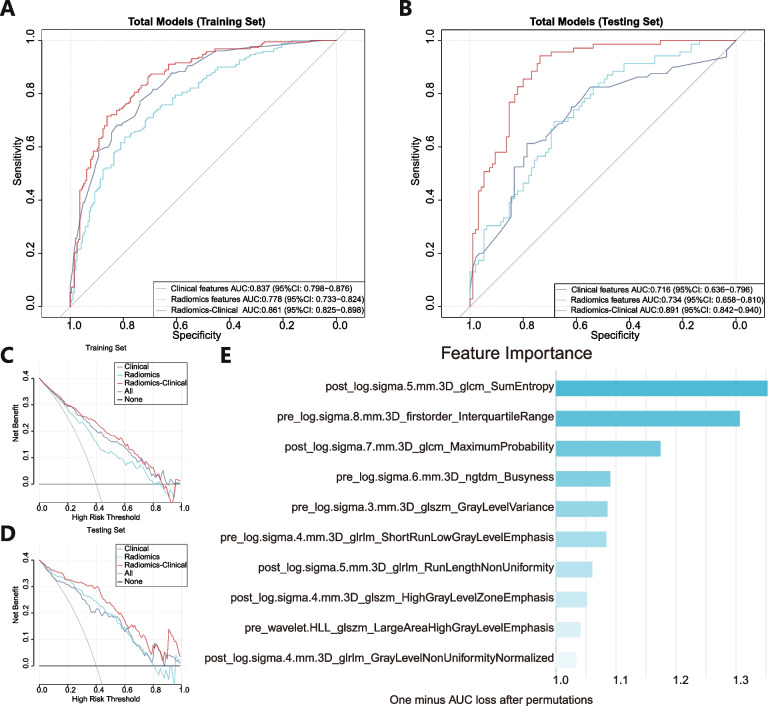
ROC curves of different models and radiomics feature importance . **A** Predictive performances of the different models in the training set. **B** Predictive performances of the different models in the testing set. DCA of predicting models in the training set (**C**) and testing set (**D**). The feature importance of the selected radiomics features in the LASSO model (**E**)

Subsequently, using different machine learning methods, we established 3 models to predict axillary pCR and compared their performance. Through logistic regression to build a clinical features model, the AUC was 0.837 in the training set and 0.716 in the testing set. The LASSO regression model from 42 radiomics features had an AUC of 0.778 in the training set and 0.734 in the testing set. However, the integrated LR radiomics-clinical combined model, which combined radiomics with clinical features, performed much better (training set AUC: 0.861, testing set AUC: 0.891) than these two single-modal models (Fig. 4A and B). The DCA also showed that the threshold probability of radiomics-clinical combined model was greater than 10% in the training set (Fig. 4C) and testing set (Fig. 4D). Feature importance of the radiomics LASSO model was analyzed. The vast majority of the features were achieved through the Laplacian of Gaussian (LoG) filter (Fig. 4E).

### Model performance in subtypes

**Fig. 5:**
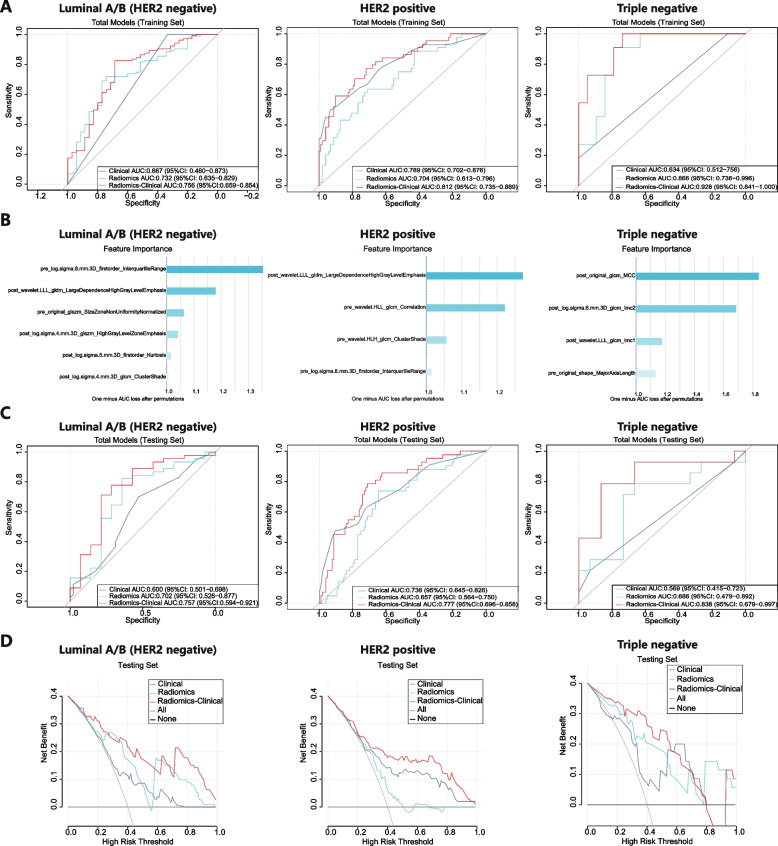
ROC curves and feature importance of different models in different subtypes. Predictive performances of the different models in the Luminal A/B (HER2 negative), HER2 positive, and Triple negative subtype in the training sets (**A**). The feature importance of the radiomics model for each subtype: Luminal A/B (HER2 negative), HER2 positive, and Triple negative (**B**). Predictive performances of the different models in each subtype in the testing sets (**C**). DCA of predicting models in each subtype in testing sets (**D**)

In subtypes analysis, as Luminal B (HER2 positive) and HER2 enriched subtype patients generally accepted the same NAC treatment, these two subtypes were pooled as a HER2 positive subtype for analysis. Then, for each subtype, we also developed 3 models to predict axillary pCR and compared their performance to determine the most optimal model. In the training sets, three radiomics-clinical combined models yielded AUCs of 0.756, 0.812, and 0.928 in Luminal A/B (HER2 negative), HER2 positive, and Triple negative subtype, respectively (Fig. 5A). Figure 5B showed all the feature importance of the selected radiomics features in Luminal A/B (HER2 negative), HER2 positive and Triple negative subtype radiomics models. In the testing sets (Fig. 5C), the radiomics-clinical combined models (AUCs: 0.757 [Luminal A/B (HER2 negative)], 0.777 [HER2 positive] and 0.838 [Triple negative]) also performed better than the clinical models (AUCs: 0.600 [Luminal A/B (HER2 negative)], 0.736 [HER2 positive] and 0.569 [Triple negative]), and the radiomics models (AUCs: 0.702 [Luminal A/B (HER2 negative)], 0.657 [HER2 positive] and 0.686 [Triple negative]). To evaluate the clinical benefit value, we used decision curve analysis to identify the model score interval that could benefit patients from model suggestions. For the Luminal A/B (HER2 negative) subtype and HER2 positive subtype, when the threshold was set more than 0.08 (Luminal A/B (HER2 negative)) and 0.13 (HER2 positive), their clinical net benefits were higher than 0 in the testing sets. However, for the Triple negative subtype, only the threshold was set at the interval of 0.04–0.56, and the clinical net benefits were higher than 0 (Fig. 5D).

### Nomogram for prediction

**Fig. 6:**
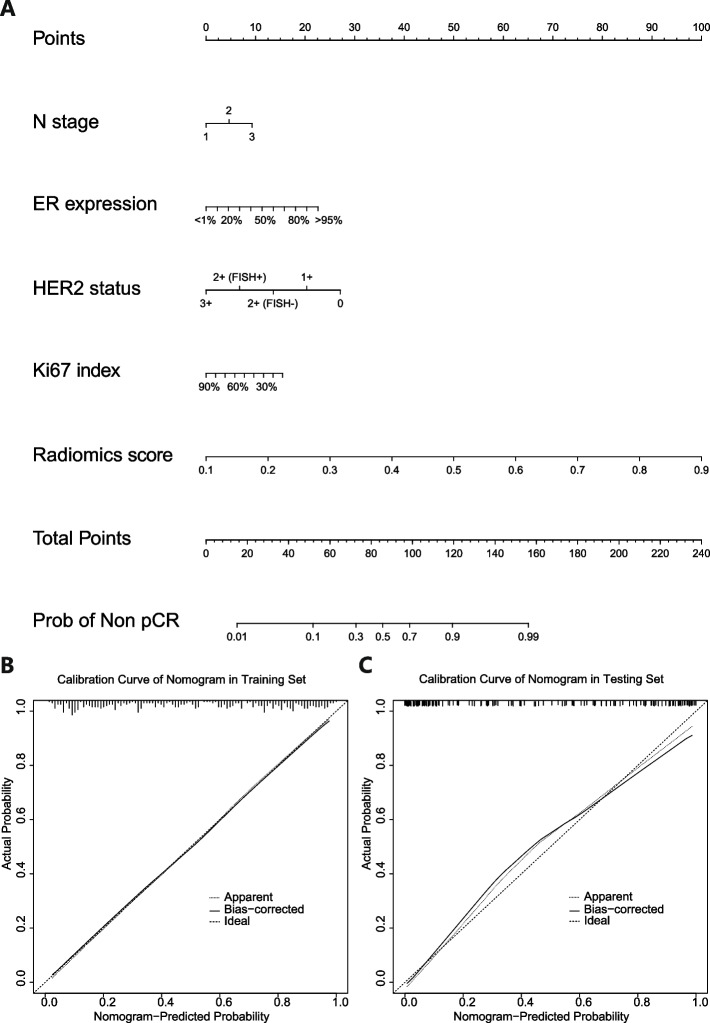
Developed the predicted nomogram based on the Combined Model in the training set (**A**). Calibration curves of nomogram in the training set (**B**) and testing set (**C**), respectively

With the results above, we developed an individualized nomogram using the LR radiomics-clinical combined model’s risk features for visualization. Then, the risk probability of axillary non-pCR for each patient could be calculated directly according to the nomogram. The calibration curves demonstrated a good agreement between the prediction probability by the nomogram and the actual observation in both the training and testing sets (see Fig. 6).

## Discussion

Accurately predicting axillary pCR is of great significance for breast cancer patients undergoing NAC. Our study found that for patients who already had axillary lymph node metastasis before NAC, using longitudinal radiomics features from pre-NAC and post-NAC CT scans, combined with clinical and pathological information, could have a good predictive effect on the patient’s axillary lymph node status.

In the past, the evaluation of axillary lymph node status after NAC always relied solely on the patient’s clinical pathological characteristics [10, 11, 19, 20]. Our study also showed that axillary pCR was related to the N stage before NAC, molecular subtype, ER/HER2 expression, and treatment regimen. However, consistent with previous reports, our logistic regression model built on baseline features cannot satisfactorily predict axillary status in the testing set (AUC = 0.716). The reason is that baseline features lack longitudinal information with NAC. In addition, baseline clinicopathological features often come from breast primary lesions, which were different from metastatic axillary lymph nodes [21]. Therefore, a model based solely on pre-NAC baseline features cannot accurately assess the post-NAC axillary lymph node status. Luckily, during the patient’s NAC process, a large amount of imaging scan data will inevitably be generated for treatment evaluation. If imaging features related to axillary lymph nodes can be extracted from these data, it will further help to improve the accuracy of the prediction model. Indeed, previous studies showed that combining clinicopathological information with radiomics could improve the performance of models [22–24]. Our results also found that building integrated prediction models through this method for axillary pCR status indeed achieved satisfactory results. The AUC value of the radiomics-clinical combined model was above 0.85 either in the training set or the testing set.

Radiomic features just from pre-NAC CT could not accurately predict axillary pCR because pre-NAC imaging data cannot cover the patient’s personalized treatment process [4]. To address this problem, this study included pre-NAC and post-NAC CT for radiomics analysis. The result showed that both pre-NAC and post-NAC could contribute valuable radiomics features for predicting axillary pCR. In the correlation analysis, we noticed that 33 pre-NAC radiomics features were significantly correlated with patients’ N stage, which means that even if the entire axilla rather than a positive lymph node was drawn as the ROI, the extracted features could also reflect the axillary metastatic burden. Although baseline data analysis showed that patients with a lower axillary metastatic burden might be more likely to achieve axillary pCR (*P* = 0.011 in the testing set), the 42 features screened by LASSO regression did not include the 33 features mentioned above. This indicated that for predicting axillary lymph node status, radiomics has specific information that is different from clinical features. The improvement in model performance by their combination also showed that radiomics features could more comprehensively reflected the post-NAC axillary lymph node status and were also independent predictors of axillary pCR as clinicopathological features.

Previous studies always used only one confirmed metastatic lymph node as the ROI for analysis and modeling [14, 15], regardless of the number of metastatic lymph nodes. However, this cannot reflect the entire metastatic burden in the axilla. It also has a problem of localization difficulty, as this single lymph node could shrink and even disappear during NAC. In this study, we creatively used the entire axilla instead of simply drawing a positive lymph node as an ROI to extract radiomics features. This can cover all imaging information in the entire axilla and even evaluate the status of infraclavicular lymph node metastasis. It also avoids the metastatic lymph node drawing difficulties and errors caused by changes after NAC. However, if using the entire axilla as an ROI, whether these obtained radiomics features could reflect total changes in these lymph nodes scattered in the axilla becomes a key issue. Fortunately, we could use different filters to solve this problem. Radiomics features are not only extracted from original images but can also be obtained from different filter-derived images. Among them, the wavelet filter could highlight texture characteristics, while the LoG filter mainly reflects grayscale change areas. So the LoG filter was often used to represent boundaries between lymph nodes and surrounding fatty tissue. And wavelet filter was used to reflect the internal texture structure of the lymph node [25]. Therefore, if drawing the entire axilla as the ROI, we speculated that using the wavelet filter could better reflect the structural differences inside enlarged axillary lymph nodes, and the LoG filter was suitable for judging the overall development of lymph node contour. In keeping with this conjecture, from pre-NAC CT images, the extracted 10 wavelet features and 9 LoG features reflected the enrichment of internal texture information and the contour increase of enlarged positive lymph nodes. Due to the drug response, enlarged lymph nodes often shrank after NAC. It was difficult to observe the internal structure of lymph nodes in detail, and only residual lymph node contours could be observed. Therefore, most of the features from post-NAC were mainly LoG filter-driven features.

For subtype analysis, due to insufficient cases, none of the models in each subtype achieved better performance than the overall case models. The stability and accuracy of these models for subtypes decreased to a certain extent. In addition, the relative increase in feature dimensions caused by the reduction of cases in subtype analysis could indeed weaken the stability of the model [26, 27]. More importantly, subtype modeling greatly weakened the value of baseline features, especially in the Luminal A/B (HER2 negative) subtype and Triple negative subtype. The pure clinical models performed the lowest AUC than radiomics models and radiomics-clinical combined models in these two subtypes. This is because the gene expression and treatment of the same subtype were always consistent. Therefore, in the same subtype, most baseline features could not distinguish treatment outcomes, and their importance was very limited in the model. Only for the HER2 positive subtype, previous studies showed that different HER2 expressions showed significantly different pCR rates [28, 29]. This also explains why the clinical model has better performance than the radiomics model in the HER2 positive subtype. The consistency also led to the result that our nomogram only screened four independent clinical features, including N stage, ER expression, HER2 status, and KI67 index. Obviously, the radiomics score had the highest score weight in the nomogram.

Our study still has some limitations. It was a retrospective single-center study lacking more prospective multicenter data to enhance its clinical value. In addition, in recent years, with the development of artificial intelligence, deep learning has been rapidly used in medical imaging [22, 30, 31]. We hope that in future research, we can add deep learning to traditional machine learning methods to achieve better results.

## Conclusion

This study successfully predicted the axillary pCR status of breast cancer patients after NAC by using pre- and post-NAC routine CT data combined with clinical pathological characteristics. This result can provide decision-making assistance for whether patients could be exempt from ALND.

## Data Availability

The datasets used and/or analyzed during the current study are available from the corresponding author upon reasonable request.
